# Ultra-Broadband Mode (De)Multiplexer on Thin-Film Lithium Niobate Platform Adopting Phase Control Theory

**DOI:** 10.3390/mi15091084

**Published:** 2024-08-28

**Authors:** Kun Yin, Wenting Jiao, Lin Wang, Shiqiang Zhu

**Affiliations:** 1 School of Mechanical Engineering, Zhejiang University, Hangzhou 310007, China; yink@zhejianglab.org; 2 Zhejiang Lab, Hangzhou 311112, China; wtjiao@zhejianglab.org

**Keywords:** thin-film lithium niobate, mode (de)multiplexer, optical phase control

## Abstract

Mode (de)multiplexers (MDMs) serve as critical foundational elements within systems for facilitating high-capacity communication, relying on mode conversions achieved through directional coupler (DC) structures. However, DC structures are challenged by dispersion issues for broadband mode coupling, particularly for high-order modes. In this work, based on the principles of phase control theory, we have devised an approach to mitigate the dispersion challenges, focusing on a thin-film lithium niobate-on-onsulator (LNOI) platform. This solution involves integrating a customized inverse-dispersion section into the device architecture, offsetting minor phase shifts encountered during the mode coupling process. By employing this approach, we have achieved broadband mode conversion from $TE_{0}$ to $TE_{1}$ and $TE_{0}$ to $TE_{2}$ within a 300 nm wavelength range, and the maximum deviations were maintained below −0.68 dB and −0.78 dB, respectively. Furthermore, the device exhibited remarkably low crosstalk, reaching down to −26 dB.

## 1. Introduction

Mode (de)multiplexing has a wide range of applications in various fields such as modern communication optics, nonlinear optics, quantum optics, and photonic integrated circuits (PICs). Especially, mode (de)multiplexers are indispensable core components of mode-division-multiplexing (MDM) communication systems, which are one of the most efficient and promising solutions to increase the communication transmission capacity against the current backdrop of exploding demand for communication technologies [1,2]. The current MDM devices are generally based on mode conversions realized by directional coupler (DC) structures [3,4,5,6], subwavelength gratings (SWGs) [7,8], multimode interferometers (MMIs) [9,10], Y-junctions [11,12], gradient index metamaterial (GIM) structures [13], etc. Among them, DC structures have been widely studied and applied for their unrivaled advantages of structural simplicity and ease of fabrication.

To satisfy the demands of high-performance WDM systems, DC structures are required to possess fundamental features including low transmission loss, broad bandwidth, and effective coupling between different modes. Given that conventional strip waveguides and rib waveguides are well suited for low-loss transmission of high-order modes, it is particularly challenging that the operating bandwidths of traditional DC devices remain limited due to dispersion issues [14].

In recent years, lithium niobate on insulator (LNOI) has emerged as an attractive material platform for PICs as a result of significant advances in the crystal ion slicing technology [15]. The LNOI platform enables high-speed electro-optical modulators and optical nonlinear devices by taking full advantage of the material properties of lithium niobate (LN), while providing a high-refractive-index contrast waveguide and dense optical confinement for high-density integration [16]. It is particularly attractive to integrate mode (de)multiplexing devices and high-speed optical modulators into the LNOI platform to realize high-capacity, low-cost PICs for applications in MDM communication systems. Less efforts have been reported for MDM devices on LNOI platforms, although there has been some research on mode (de)multiplexers based on DC [15,17], yet all of them are facing the same bandwidth limitation problem mentioned above.

In this work, based on the principles of the phase control theory, we have devised an approach to mitigate the dispersion challenges, focusing on the LNOI platform, which offers advantages of broadband (operating over a wide range of wavelength), high conversion efficiency, low crosstalk, and ease of fabrication. This solution involves integrating a customized inverse-dispersion section into the device architecture, offsetting minor phase shifts encountered during the mode coupling process. By employing this approach, the results show that the mode converters of $TE_{0}$ to $TE_{1}$ and $TE_{0}$ to $TE_{2}$ proposed in this article can realize a conversion efficiency of 86% and 88% at 1550 nm, as well as not less than 57% within an ultra-wide operating bandwidth of 300 nm. The cascade structure can realize the multiplexing and demultiplexing of multiple modes. These findings highlight an exceptionally wideband, high-efficiency mode (de)multiplexing approach, which is adaptable for scaling to support more modes and diverse material platforms. This advancement offers an important guide for designing core components in mode (de)multiplexing within photonic integrated circuits (PICs), with significant implications for applications in laser-driven accelerators, communication systems, and optical computing.

## 2. Device Design and Method

### 2.1. Structure and Theoretical Analysis

**Figure 1:**
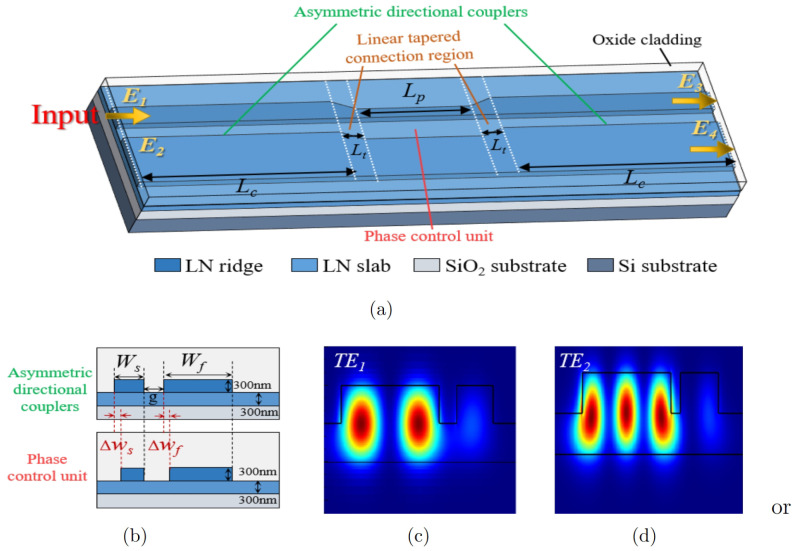
(**a**) Schematic of the proposed broadband mode converter. (**b**) Cross-sectional views of the asymmetric directional couplers and the phase control unit. (**c**) Mode field distribution of $TE_{1}$ mode. (**d**) Mode field distribution of $TE_{2}$ mode.

Our broadband mode multiplexers are based on the designed broadband mode converter, which is built up for a 600 nm thick film of LN, with a 300 nm etch depth to create the ridge waveguide. As shown in Figure 1a, the proposed device consists of two asymmetric directional couplers with the same structure, between which a phase control unit is then inserted. A linear tapered connection region is used to connect the phase control unit to its left and right asymmetric directional couplers. Each asymmetric directional coupler is composed of a single-mode waveguide and a few-mode waveguide of widths $W_{s}$ and $W_{f}$ with a gap *g* in between, as can be seen from Figure 1b. The phase control unit comprises a thin waveguide and a wide waveguide separated by a gap, wherein the thin waveguide has a width of $W_{s}-\Delta w_{s}$ and the wide waveguide has a width of $W_{f}-\Delta w_{f}$. The cross-sectional views of the asymmetric directional couplers and the phase control unit are shown in the upper and lower halves of Figure 1b. The linear tapered connection region has a length of 2 μm each. The lengths of the two asymmetric directional couplers and the phase control unit are defined as $L_{c}$ and $L_{p}$, respectively. The mode field distributions of $TE_{1}$ mode and $TE_{2}$ mode are illustrated in Figure 1c,d with different waveguide widths.

As shown in Figure 1a (marked with yellow arrows), the fundamental mode light ($TE_{0}$ mode) is emitted into the input port on the left side of the broadband mode converter. When the fundamental mode light propagates along the asymmetric directional coupler on the left side, if the fundamental mode in the single-mode waveguide satisfies the phase-matching condition with the high-order mode in the few-mode waveguide (lower waveguide), the fundamental mode can be converted to the high-order mode and coupled into the few-mode waveguide for transmission, of which the conversion efficiency is wavelength-dependent. When passing through the phase control unit, the light propagated in the thin waveguide and the wide waveguide produce a small relative phase shift. After that, the fundamental mode is converted to the high-order mode again in the asymmetric directional coupler on the right side under the satisfaction of the phase matching condition, and then it is finally output. Moreover, the fundamental mode directly input from the few-mode waveguide can pass through the asymmetric directional coupling region and the phase control region without interference, thereby enabling the mode multiplexing function for the simultaneous transmission of the fundamental mode and the high-order mode in the few-mode waveguide. The inverse process of light propagation described above is demultiplexing.

Hereafter, we theoretically analyze the proposed broadband mode converter using the transfer matrix method (TMM). The relationship between the input and output electric fields of the mode converter can be expressed as follows [18]:

(1) $$\left[\begin{array}{c} E_{3}\\ E_{4} \end{array}\right]=C_{a}\cdot T\cdot P\cdot T^{-1}\cdot C_{a}\cdot \left[\begin{array}{c} E_{1}\\ E_{2} \end{array}\right],$$

where $E_{1}$ and $E_{2}$ are the electric fields at the inputs, and $E_{3}$ and $E_{4}$ represent the electric fields at the outputs. As can be seen in Figure 1a, the mode converter has only one input; therefore, the value $E_{2}=0$ can be obtained. $C_{a}$ is the coupling matrix of the two asymmetric directional couplers, and *P* describes the propagation matrix of the phase control unit. The propagation matrices of the linear tapered connection region located to the right and left of the phase control unit are, respectively, denoted by *T* and $T^{-1}$, where $T^{-1}$ is the inverse matrix of *T*.

According to the principle of the TMM [19], the expression of the matrix $C_{a}$ can be calculated as

(2) $$C_{a}=\left[\begin{array}{cc} t_{f}\varphi_{f}\; & -jk_{h}\varphi_{h}\\ -jk_{f}\varphi_{f}\; & t_{h}\varphi_{h} \end{array}\right]\cdot exp\left(-\frac{\alpha_{a}}{2}L_{c}\right)$$

where $t_{f,h}$ denote the straight-through coefficients of the asymmetric directional couplers, and $k_{f,h}$ are cross-coupling coefficients. It should be explained that the same variables corresponding to the fundamental mode and the high-order mode are distinguished by footnotes *f* and *h*, respectively. The structures of the asymmetric directional couplers under consideration are reciprocal. Therefore,

(3) $$t_{f}^{2}+k_{f}^{2}=1\;;\;t_{h}^{2}+k_{h}^{2}=1$$

$\varphi_{f,h}$ are the phase shifts produced by the coupling and propagation of the fundamental mode and the high-order mode in the asymmetric directional couplers, which have the following expression:

(4) $$\varphi_{f,h}=e^{-j\frac{2\pi }{\lambda }\cdot n_{eff-pm}\cdot L_{c}}$$

where λ is the wavelength, and $L_{c}$ is the length of the asymmetric directional coupler. $n_{eff-pm}$ is the effective index of the fundamental mode and the high-order mode under the phase-matching condition in the asymmetric directional couplers.

**Figure 2:**
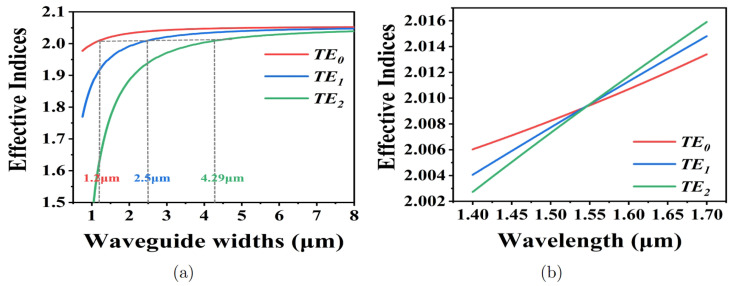
(**a**) Effective indices for $TE_{0}$ mode, $TE_{1}$ mode, and $TE_{2}$ mode at different waveguide widths. (**b**) Effective indices as a function of wavelength when waveguide widths are equal to 1.2 μm, 2.5 μm, and 4.29 μm, respectively, for $TE_{0}$ mode, $TE_{1}$ mode, and $TE_{2}$ mode.

Effective indices calculated for the $TE_{0}$ mode, $TE_{1}$ mode, and $TE_{2}$ mode at different waveguide widths when the modes are transmitted in the rib waveguide on the LNOI platform are illustrated in Figure 2a. As indicated by the gray dashed line in Figure 2a, the effective index of the $TE_{0}$ mode transmitted in a 1.2 μm wide waveguide is equal to that of the $TE_{1}$ mode in a 2.5 μm wide waveguide as well as that of the $TE_{2}$ mode in a 4.29 μm wide waveguide, respectively. This means that the phase-matching conditions are basically satisfied. It must be noted that the waveguide widths under phase-matching conditions corresponding to the different modes obtained here are not absolutely accurate. Figure 2b exhibits effective indices for the different modes as a function of wavelength. We assume that propagation losses of all the waveguides in the proposed device are the same and denoted as $\alpha_{a}$.

The propagation matrix of the phase control unit, *P*, is given by

(5) $$P=\left[\begin{array}{cc} \varphi_{fp}\cdot exp\left(-\frac{\alpha_{a}}{2}L_{p}\right)\; & 0\\ 0\; & \varphi_{hp}\cdot exp\left(-\frac{\alpha_{a}}{2}L_{p}\right) \end{array}\right]$$

where $L_{p}$ is the length of the phase control unit, and $\varphi_{fp}$ and $\varphi_{hp}$ denote phase shifts produced by the fundamental as well as the high-order modes as they pass through the phase control unit, respectively. $\varphi_{fp}$ and $\varphi_{hp}$ can be written as follows:

(6) $$\varphi_{fp}=e^{-j\frac{2\pi n_{f}}{\lambda }L_{p}}\;;\;\varphi_{hp}=e^{-j\frac{2\pi n_{h}}{\lambda }L_{p}}$$

$n_{f}$ is the effective index of the fundamental mode primarily confined in the thin waveguide of the phase control unit, and $n_{h}$ is the effective index of the high-order mode primarily confined in the wide waveguide. $n_{f,h}$ can be essentially determined from Figure 2a.

Additionally, we have assumed that *T* can be approximated by the following:

(7) $$T=\left[\begin{array}{cc} exp\left(-j\theta_{f}-\frac{\alpha_{a}}{2}L_{t}\right)\; & 0\\ 0\; & exp\left(-j\theta_{h}-\frac{\alpha_{a}}{2}L_{t}\right) \end{array}\right]$$

where $\theta_{f,h}$ are the phase shifts of the tapered waveguide in the linear tapered connection region. $L_{t}$ is the length of the linear tapered connection region, which is 2 μm in our design. We determined $\theta_{f,h}$ as a function of wavelength using FDTD Solutions by Lumerical Solutions, Inc. (Vancouver, BC, Canada) [20].

Our proposed broadband mode converter, as can be seen in Figure 1a, has $E_{1}$ as the input electric field when $E_{2}=0$. Then, the conversion efficiency is defined here as

(8) $$\nu_{c}=\frac{{\left|E_{4}\right|}^{2}}{{\left|E_{1}\right|}^{2}}$$

**Figure 3:**
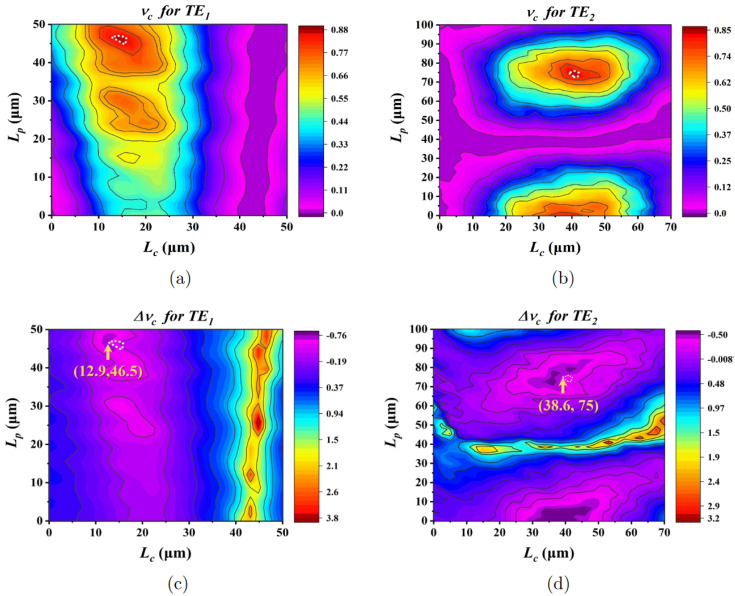
Contour maps of $\nu_{c}$ as functions of $L_{c}$ and $L_{p}$ at λ =1550 nm for the mode converter of (**a**) $TE_{0}$ mode to $TE_{1}$ mode and (**b**) $TE_{0}$ mode to $TE_{2}$ mode. Contour maps of $\Delta \nu_{c}$ as functions of $L_{c}$ and $L_{p}$ in the wavelength range from 1400 nm to 1700 nm for the mode converter of (**c**) $TE_{0}$ mode to $TE_{1}$ mode and (**d**) $TE_{0}$ mode to $TE_{2}$ mode.

Using Equation (8), it is very convenient to find out the desired values of $L_{c}$ and $L_{p}$ corresponding to a mode multiplexer with broad operation bandwidth and high conversion efficiency. Figure 3a,b plot the contour maps of $\nu_{c}$ at a wavelength of 1550 nm with a variation in $L_{c}$ and $L_{p}$ as $W_{s}=1.2\;\mu$m, $\Delta w_{s}=\Delta w_{f}=0.3\;\mu$m, and g=0.3μm. Additionally, $W_{f}$ are equal to 2.5μm for $TE_{1}$ mode and 4.29μm for $TE_{2}$ mode, respectively. In order to obtain desired $\nu_{c}$ with a large operating bandwidth, we further plot a maximum deviation of $\nu_{c}$ as functions of $L_{c}$ and $L_{p}$ in a 300 nm wavelength range centered at 1550 nm, as shown in Figure 3c,d. The maximum deviation of $\nu_{c}$ is defined as

(9) $$\Delta \nu_{c}={\left\{\mathrm{lg}\left(\frac{\nu_{c}\left(1550\;\mathrm{nm}\right)-\nu_{c}\left(\lambda \right)}{\nu_{c}\left(1550\;\mathrm{nm}\right)}\right)\right\}}_{max}$$

For the purpose of deriving the optimal $L_{c}$ and $L_{p}$ that satisfy both high $\nu_{c}$ and small $\Delta \nu_{c}$ conditions, it is useful to start with a region in the contour maps of $\nu_{c}$ that meets the expectation (e.g., the regions circled by the white dashed line in Figure 3a,b) and then find the overlap between the regions and the small $\Delta \nu_{c}$ regions in the contour maps of $\Delta \nu_{c}$. As shown Figure 3c,d, the arrow indicates that the optimal parameters are selected to be $L_{c}=12.9\;\mu$m and $L_{p}=46.5\;\mu$m for $TE_{1}$ mode and selected to be $L_{c}=38.6\;\mu$m and $L_{p}=75\;\mu$m for $TE_{2}$ mode. As can be seen from Figure 3, the conversion efficiency $\nu_{c}$ is not less than 85% for both $TE_{0}$ to $TE_{1}$ and $TE_{0}$ to $TE_{2}$, and the maximum deviation $\Delta \nu_{c}$ can be maintained below −0.76 dB ($TE_{1}$) and −0.5 dB $TE_{2}$ in the wavelength range of 300 nm.

### 2.2. Three-Dimensional FDTD Simulation

**Figure 4:**
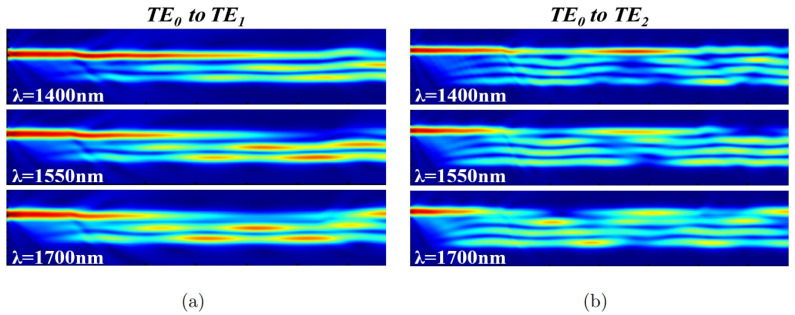
Power distributions in the plane of z=0 of the broadband mode converters for (**a**) $TE_{0}$ mode to $TE_{1}$ mode and (**b**) $TE_{0}$ mode to $TE_{2}$ mode at different wavelengths.

The above TMM theoretical calculations provide a basic range of $L_{c}$, $L_{p}$, $\Delta w_{s}$, and $\Delta w_{f}$ for the optimum conversion efficiency. The more refined three-dimensional time-domain finite-difference (3D FDTD) method is used in the next step for further simulation and optimization of the whole device. Here, as an example, we give the simulation results of the broadband mode converters for $TE_{0}$ to $TE_{1}$ and $TE_{0}$ to $TE_{2}$, respectively. Figure 4a,b display the power distributions for the broadband mode converters at three different wavelengths in the plane of z=0. The optimal device dimensions for $TE_{0}$ to $TE_{1}$ mode converter are $L_{c}=11.9\;\mu$m, $L_{p}=47.1\;\mu$m, $W_{s}=1.2\;\mu$m, $W_{f}=2.5\;\mu$m, $\Delta w_{s}=0.37\;\mu$m, and $\Delta w_{f}=0.37\;\mu$m; for the $TE_{0}$ to $TE_{2}$ mode converter, these are $L_{c}=36.6\;\mu$m, $L_{p}=76.9\;\mu$m, $W_{s}=1.2\;\mu$m, $W_{f}=4.29\;\mu$m, $\Delta w_{s}=0.31\;\mu$m, and $\Delta w_{f}=0.1\;\mu$m. The waveguide gap g=0.3μm.

**Figure 5:**
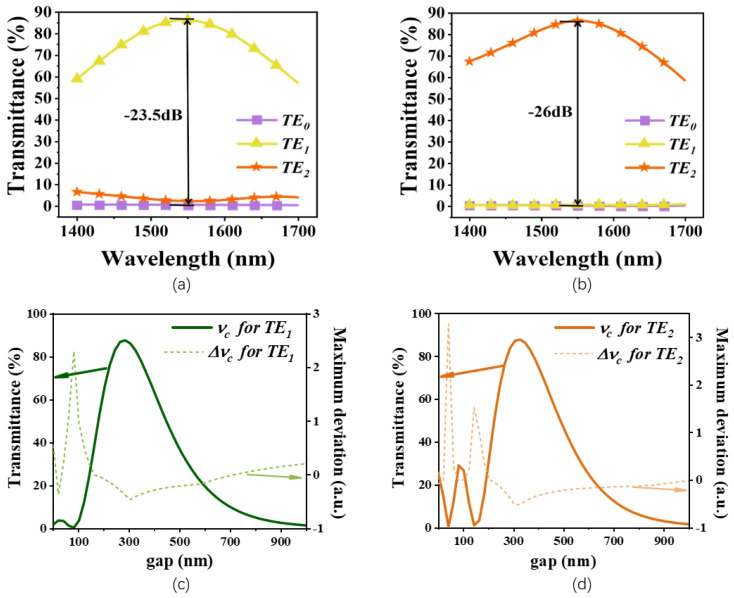
The calculated crosstalk of the broadband mode converters for (**a**) $TE_{0}$ mode to $TE_{1}$ mode and (**b**) $TE_{0}$ mode to $TE_{2}$ mode. The influence of gap change between thin waveguide and wide waveguide for (**c**) $TE_{0}$ − $TE_{1}$ and (**d**) $TE_{0}$ − $TE_{1}$.

Crosstalk between different modes is an important parameter for evaluating the performance of mode (de)multiplexing devices. Figure 5 gives the transmittance of different modes in the mode converters for $TE_{0}$ mode to $TE_{1}$ mode and $TE_{0}$ mode to $TE_{2}$ mode with the corresponding crosstalk calculated. It can be seen from the results that the conversion efficiency reaches 86% and 88% for a wavelength of 1550 nm, while the inter-modal crosstalk from adjacent order modes is −23.5 dB and −26 dB for $TE_{1}$ and $TE_{2}$ modes at the wavelength of 1550 nm, respectively. One hundred μm long taper waveguides are used to connect multimode waveguides with different widths to reduce the inter-modal crosstalk. We scanned the effect of varying the gap between the thin and wide waveguides on the mode conversion efficiency and wavelength dependence while keeping other parameters fixed, as shown in Figure 5c,d. We observed that a gap of 300 nm yielded the optimal results for both the mode conversion efficiency and wavelength correlation. Theoretically, a smaller gap would lead to a higher coupling efficiency and potentially allow for a more compact device size. However, due to the low etching rate of LN and the significant impact of the loading effect, gaps smaller than 300 nm often result in considerable deviations in the etching depth. Therefore, we chose a gap of 300 nm to achieve consistency between the simulation and experimental results.

**Figure 6:**
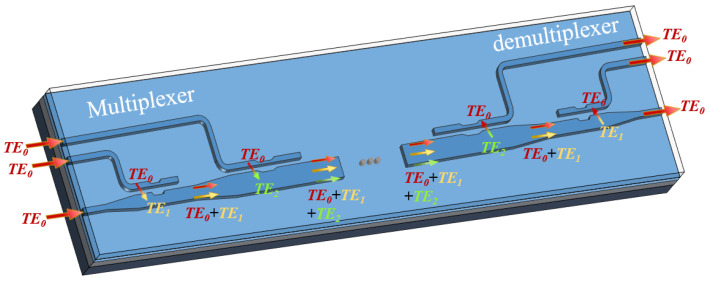
Illustrative of the $TE_{0}+TE_{1}+TE_{2}$ (de)multiplexer concept.

Additionally, in a future work, this technique can be extended to achieve multimode multiplexing or demultiplexing by connecting the mode converters obtained through the aforementioned design method. For illustrative purposes, the combination of multiplexed signals $TE_{0}+TE_{1}+TE_{2}$ is demonstrated in Figure 6. Using the mode multiplexer as an example, all three ports on the left side are fed with fundamental mode signal $TE_{0}$, of which two fundamental mode signals are converted to $TE_{1}$ and $TE_{2}$ modes, respectively, via two phase-control assisted mode converters, and sequentially enter into the lowest bus waveguide and converge with the $TE_{0}$ mode that is originally transmitted in it. The multiplexed signals ($TE_{0}+TE_{1}+TE_{2}$) continue forward for the next optical processing and are then demultiplexed.

For a comparison, Table 1 summarizes the performance of previously reported mode convertors simulated or experimented on different material platforms. It can be seen that this work achieves an ultra-wide operating bandwidth, while the conversion efficiency can still maintain high values. The lowest crosstalk shows the potentially excellent performance of this work in the case of multimode multiplexing operation.

For a comparison, Table 1 provides a comprehensive summary of the performance metrics of previously reported mode converters on LNOI, including both simulated and experimentally validated devices. This comparison highlights the unique advantages of our approach. Our work achieves an ultra-wide operating bandwidth, which is crucial for a broad range of applications in PICs. Despite the extensive bandwidth, the conversion efficiency remains impressively high, showcasing the robustness and effectiveness of our design. Additionally, the exceptionally low crosstalk observed in our results suggests that this work has the potential to deliver outstanding performance in scenarios involving multimode multiplexing operations. This aspect is particularly important for advanced applications such as laser-driven accelerators, high-speed communications, and optical computing, where minimizing interference between modes is critical for maintaining signal integrity and overall system efficiency.

## 3. Conclusions

We have demonstrated a ultra-broadband, high-efficiency mode (de)multiplexer on a thin-film lithium niobate platform for $TE_{0}$ to $TE_{1}$ and $TE_{0}$ to $TE_{2}$ whilst adopting the phase control theory. The results show that the mode converters of $TE_{0}$ to $TE_{1}$ and $TE_{0}$ to $TE_{2}$ proposed in this article can realize a conversion efficiency of 86% and 88% at 1550 nm, as well as not less than 57% within an ultra-wide operating bandwidth of 300 nm. The inter-modal crosstalk from adjacent order modes is −23.5 dB and −26 dB for $TE_{1}$ and $TE_{2}$ modes at a wavelength of 1550 nm, respectively. Utilizing the proposed design methodology based on the TMM combined with the contour maps, it is feasible to obtain mode converters with large operating bandwidths rapidly. Mode (de)multiplexing devices with wide bandwidths can be easily developed by cascading mode converters, such as $TE_{0}+TE_{1}+TE_{2}$, as demonstrated. This approach has the potential to be extended to the multiplexing of additional modes. Moreover, although the mode (de)multiplexing devices demonstrated in this article are based on the LNOI platform, it is also possible to realize large operating bandwidth mode multiplexing on other photoelectric material platforms using the approach we introduced in Section 2.

Overall, the results demonstrate an ultra-broadband, high-efficiency mode (de)multiplexing technique that can be scaled to accommodate additional modes and various material platforms. This discovery provides a valuable reference for the design of fundamental components in mode (de)multiplexing within photonic integrated circuits (PICs), with particular relevance to applications in laser-driven accelerators, communications, and optical computing.

## Figures and Tables

**Table 1:** Summary of reported mode convertors on LNOI.

Reference	Waveguide	Conversion Efficiency (%)	Crosstalk (dB)	Bandwidth (nm)
[21]	polymer-loaded LNOI	40% (ex.)	−9.5	70
[15]	silicon nitride loaded LNOI	86.5% (ex.)	−14.7	40
[22]	LNOI strip	34% (ex.)	−10.6	>130
this work	LNOI ridge	88% (si.)	−26.0	300

ex.: experiment; si.: simulation

## Data Availability

Data is contained within the article.

## References

[1] Khonina S.N., Kazanskiy N.L., Butt M.A., Karpeev S.V. (2022). Optical multiplexing techniques and their marriage for on-chip and optical fiber communication: A review. Opto-Electron. Adv..

[2] Wang J., Long Y. (2018). On-chip silicon photonic signaling and processing: A review. Sci. Bull..

[3] Chen Z., Zhu Y., Ruan X., Li Y., Li Y., Zhang F. (2018). Bridged Coupler and Oval Mode Converter Based Silicon Mode Division (De)Multiplexer and Terabit WDM-MDM System Demonstration. J. Light. Technol..

[4] Ding Y., Xu J., Ros F.D., Huang B., Ou H., Peucheret C. (2013). On-chip two-mode division multiplexing using tapered directional coupler-based mode multiplexer and demultiplexer. Opt. Express.

[5] Zhang Z., Hu X., Wang J. (2015). On-chip optical mode exchange using tapered directional coupler. Sci. Rep..

[6] Huang Q., Wu Y., Jin W., Chiang K.S. (2018). Mode Multiplexer With Cascaded Vertical Asymmetric Waveguide Directional Couplers. J. Light. Technol..

[7] Sun Q., Chen H., Wang J., Yang J., Jia H. (2024). Broadband mode-division (de) multiplexer using nanorod-assisted multimode subwavelength gratings. Opt. Commun..

[8] González-Andrade D., Dias A., Wangüemert-Pérez J.G., Ortega-Moñux A., Molina-Fernández I., Halir R., Cheben P., Velasco A.V. (2020). Experimental demonstration of a broadband mode converter and multiplexer based on subwavelength grating waveguides. Opt. Laser Technol..

[9] Uematsu T., Ishizaka Y., Kawaguchi Y., Saitoh K., Koshiba M. (2012). Design of a Compact Two-Mode Multi/Demultiplexer Consisting of Multimode Interference Waveguides and a Wavelength-Insensitive Phase Shifter for Mode-Division Multiplexing Transmission. J. Light. Technol..

[10] González-Andrade D., Olivares I., de Cabo R.F., Vilas J., Dias A., Velasco A.V. (2023). Broadband three-mode converter and multiplexer based on cascaded symmetric Y-junctions and subwavelength engineered MMI and phase shifters. Opt. Laser Technol..

[11] Chang W., Lu L., Ren X., Li D., Pan Z., Cheng M., Liu D., Zhang M. (2018). Ultra-compact mode (de) multiplexer based on subwavelength asymmetric Y-junction. Opt. Express.

[12] Chen W., Wang P., Yang T., Wang G., Dai T., Zhang Y., Zhou L., Jiang X., Yang J. (2016). Silicon three-mode (de)multiplexer based on cascaded asymmetric Y junctions. Opt. Lett..

[13] He Y., Li X., Zhang Y., An S., Wang H., Wang Z., Chen H., Huang Y., Huang H., Fontaine N.K. (2023). On-chip metamaterial-enabled high-order mode-division multiplexing. Adv. Photonics.

[14] Li C., Liu D., Dai D. (2019). Multimode silicon photonics. Nanophotonics.

[15] Han X., Jiang Y., Frigg A., Xiao H., Zhang P., Nguyen T.G., Boes A., Yang J., Ren G., Su Y. (2022). Mode and polarization-division multiplexing based on silicon nitride loaded lithium niobate on insulator platform. Laser Photonics Rev..

[16] Chen G., Li N., Ng J.D., Lin H.L., Zhou Y., Fu Y.H., Lee L.Y.T., Yu Y., Liu A.Q., Danner A.J. (2022). Advances in lithium niobate photonics: Development status and perspectives. Adv. Photonics.

[17] Zhang M., Ai W., Chen K., Jin W., Chiang K.S. (2018). A Lithium-Niobate Waveguide Directional Coupler for Switchable Mode Multiplexing. IEEE Photonics Technol. Lett..

[18] Lukas C., Hochberg M. (2015). Silicon Photonics Design: From Devices to Systems.

[19] Mackay T.G., Lakhtakia A. (2020). The Transfer-Matrix Method in Electromagnetics and Optics.

[20] https://www.lumerical.com/.

[21] Yu Z., Tong Y., Tsang H.K., Sun X. (2020). High-dimensional communication on etchless lithium niobate platform with photonic bound states in the continuum. Nat. Commun..

[22] Zhu W., Deng C., Wang D., Wang Q., Sun Y., Wang J., Yun B., Cui Y., Hu G. (2024). Four-Channel Broadband Mode (De) multiplexer Based on Thin-Film Lithium Niobate Platform. ACS Photonics.

